# Polymer Brush in a Nanopore: Effects of Solvent Strength and Macromolecular Architecture Studied by Self-Consistent Field and Scaling Theory

**DOI:** 10.3390/polym13223929

**Published:** 2021-11-14

**Authors:** Mikhail Y. Laktionov, Ekaterina B. Zhulina, Ralf P. Richter, Oleg V. Borisov

**Affiliations:** 1 Petersburg National Research University of Information Technologies, Mechanics and Optics, 197101 St. Petersburg, Russia; miklakt@gmail.com (M.Y.L.); kzhulina@hotmail.com (E.B.Z.); 2 Institute of Macromolecular Compounds of the Russian Academy of Sciences, 199004 St. Petersburg, Russia; 3 University of Leeds, School of Biomedical Sciences, Faculty of Biological Sciences, School of Physics and Astronomy, Faculty of Engineering and Physical Sciences, Astbury Centre for Structural Molecular Biology, and Bragg Center for Materials Research, Leeds LS2 9JT, UK; R.Richter@leeds.ac.uk; 4 CNRS, Université de Pau et des Pays de l’Adour UMR 5254, Institut des Sciences Analytiques et de Physico-Chimie pour l’Environnement et les Matériaux, 64053 Pau, France

**Keywords:** polymer brushes, nanopores, conformational transitions, pore opening/closing transition

## Abstract

To study conformational transition occuring upon inferior solvent strength in a brush formed by linear or dendritically branched macromolecules tethered to the inner surface of cylindrical or planar (slit-like) pore, a self-consistent field analytical approach is employed. Variations in the internal brush structure as a function of variable solvent strength and pore radius, and the onset of formation of a hollow channel in the pore center are analysed. The predictions of analytical theory are supported and complemented by numerical modelling by a self-consistent field Scheutjens–Fleer method. Scaling arguments are used to study microphase segregation under poor solvent conditions leading to formation of a laterally and longitudinally patterned structure in planar and cylindrical pores, respectively, and the effects of confinement on "octopus-like" clusters in the pores of different geometries.

## 1. Introduction

Polymer brushes are layers of macromolecules tethered by terminal segments to a solid substrate and immersed in a solvent [1,2,3,4,5]. Grafting of macromolecules to planar substrates or to the surface of colloidal particles gives rise to planar or colloidal polymer brushes, respectively. If the solubility of the brush forming macromolecules can be tuned by varying environmental conditions (e.g., temperature), adhesive, tribological, and biointeractive properties of the substrates and colloidal stability of dispersions can be controlled through conformational changes (i.e., swelling-to-collapse) in the brush [6,7,8,9,10]. A novel trend in the molecular design of smart polymer-modified interfaces assumes that the topological diversity of the brush forming macromolecules that enables tuning of the response functions with respect to external stimuli through varied macromolecular architecture can be exploited [11,12,13].

The grafting of brushes composed of stimuli-responsive polymers onto the inner surface of nano- or mesoscopic pores in porous materials allows for controlled partitioning of different molecules between the pores and the bulk solution in which the porous medium is immersed [14,15,16]. Furthermore, decorating walls of nanopores that perforate a membrane may ensure selective and controlled permeability of the membrane for (bio)molecules [17]. The principles of “permselectivity” can be borrowed by biomimetic nanotechnology from nuclear pore complexes (NPCs) that control bulk macromolecular exchange between the cytoplasm and the nucleus of eukaryotic cells. In their central channel, which measures tens of nanometers in diameter, NPCs harbour intrisically disordered and thus flexible protein domains (FG domains) that are grafted to the channel walls and rich in phenylalanine-glycine (FG) motifs thus being moderately hydrophobic [18,19,20]. Since partitioning in and transport through polymer decorated nanopores can be regulated by conformational changes in the brush, it is important to understand the pecularities of the swelling-to-collapse conformational transitions occuring in the polymer brush tethered to the inner surface of the pores as a function of the solvent quality.

The theory of the swelling-to-collapse conformational transition in planar brushes of linear polymers was developed within strong stretching self-consistent field approximation in References [21,22] and then generalised for brushes formed by dendritically branched macromolecules in Reference [23]. A similar approach was applied [24] to concave (curved inwards) brushes of linear chains swollen in a good solvent.

The aim of the present paper is to study conformational transitions in brushes formed by polymers with arbitrary (linear or tree-like) architecture grafted to the inner surface of cylindrical or slit-like pores and immersed into a solvent of arbitrary quality. Our emphasis lies on an analysis of the evolution of the intra-pore polymer density profiles and the pore filling/opening threshold as a function of variable solvent strength and the architecture of brush forming macromolecules. To this end, we employ the analytical strong-stretching self-consistent field (SS-SCF) approximation (Section 2) and complement it by numerical modelling based on a self-consistent field Scheutjens–Fleer method. The latter does not involve approximations of strong stretching of the brush forming chains and enable us to investigate the effects of polymer density fluctuations near the edge of the brush. Whilst the brush is laterally or longitudinally uniform under good or moderately poor solvent conditions (when the chains are stretched), it splits into an array of finite-size clusters upon a decrease in solvent strength, i.e., when the chains lose stretching beyond Gaussian dimensions. The interplay between brush confinement and clusters formation is analysed in Section 3 using a scaling approach. The Conclusions are formulated in Section 4.

## 2. Self-Consistent Field Theory for Polymer and Dendron Brushes in the Pore

### 2.1. Analytical Theory: Strong-Stretching Approximation

**Figure 1:**
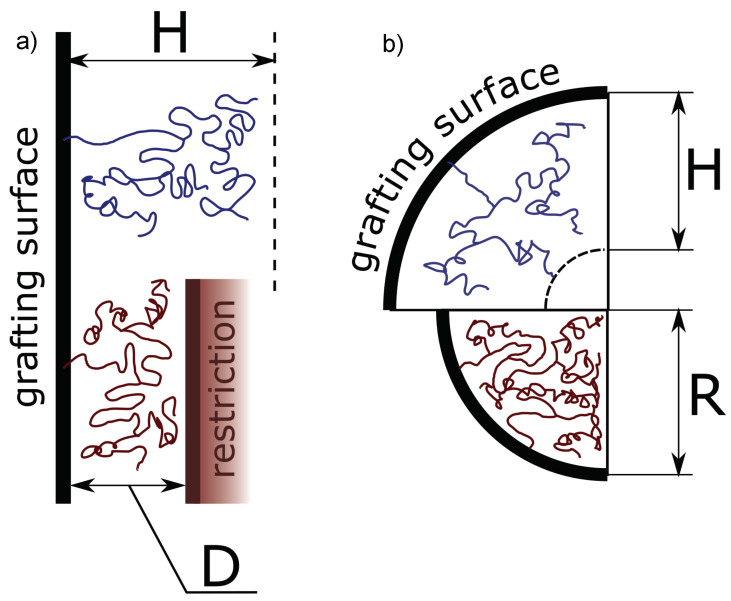
Schematics of a brush formed by first generation dendrons grafted on a planar surface (**a**) or to the inner surface of a cylindrical pore of radius *R*. In the scenarios depicted with a red polymer chain, the brush pervades the entire space of a planar slit (**a**) or the cylindical pore (**b**).

We consider a brush formed by long flexible polymer chains with degree of polymerisation (DP) *N* and arbitrary linear or tree-like (dendron) architecture, grafted by one end to the inner surface of a cylindrical pore of radius *R* and immersed in a solvent (Figure 1). Here and below, we assume the brush forming chains (or any linear segment of the them) to be intrinsically flexible. Each monomer unit is assumed to have a length *a* and a volume $a^{3}$, and in the following, all dimensions are normalised by the monomer unit length (approximately equal to the statistical segment length). The pore is assumed to be long so that edge effects on the conformation of the brush forming macromolecules are disregarded. For comparison, we also consider the case of a slit-like pore of thickness 2D.

The grafting density σ=1/s is related to the area per chain *s* or number of chains 1/h per unit length of the pore,

(1) $$\sigma =\frac{1}{2\pi Rh}$$

Obviously, the pore can accomodate a polymer brush if $R\geq R_{min}$, where $R_{min}={\left(N/\pi h\right)}^{1/2}=2N\sigma$.

The solvent quality is characterised by the Flory–Huggins parameter χ. In our recent paper [23], we considered the collapse of a brush formed by linear or branched macromolecules in the case when the pore radius exceeds the brush thickness *H*, i.e, R≥H, and there is a hollow channel around the pore axis. Here, we focus primarily on the case when, under good solvent conditions, the brush filles the pore, that is, the polymer density is nonzero at z∈[0,R], where *z* is the distance from the wall towards the pore axis. The transition from R≤H (filled pore) to R≥H (open pore) triggered by variations of the solvent strength (i.e., in parameter χ) or of the pore radius *R* are analysed.

An analytical strong-stretching self-consistent field (SS-SCF) approach formulated initially for brushes of linear chains [21,25] and later extended to brushes of branched polymers [26,27,28,29] presumes linear (Gaussian) entropic elasticity of any linear segment of the brush forming macromolecules and operates with the chain trajectories z(m) that specify the most probable position *z* of the monomer unit with ranking number *m* with respect to the grafting surface.

Within the strong-stretching self-consistent field approximation monomer units in the brush are subjected to the self-consistent molecular potential that exhibits a parabolic dependence on the distance *z* from the grafting surface [29]

(2) $$\frac{\partial f\{\phi (z)\}}{\partial \phi (z)}=\frac{3}{2}\kappa^{2}\left(\Lambda^{2}-z^{2}\right)$$

where ϕ(z) is the volume fraction of monomer units in the brush, f{ϕ(z)} is the free energy of interactions in the brush per unit volume, κ is a coefficient dependent on the DP and topology of the brush forming chains, and the parameter Λ is specified below. For linear chains, κ=π/2N, whereas for tree-like or cycle-containing polymers,

(3) $$\kappa =\frac{\pi \eta }{2N}$$

where η≥1 is the so-called topological ratio that can be calculated for particular macromolecular architectures (tree-like or cycled) following earlier developed routines [29,30]. The topological ratio quantifies relative increases in the conformational entropy losses in brushes formed by branched (or cycled) polymers compared with those in brushes of linear chains with the same DP.

Equation (2) presumes Gaussian (linear) conformational elasticity of the brush-forming chains on all the length scales and absence of “dead zones” depleted of the chain ends proximal to the grafting surface. Remarkably, Equation (2) is applicable irrespective of the specific type of interactions (functional form of f{ϕ(z)}) in the brush.

Here, we apply the mean field Flory–Huggins approximation

(4) $$\frac{f\{\phi (z)\}}{k_{B}T}=\left(1-\phi \left(z\right)\right)\ln\left(1-\phi \left(z\right)\right)+\chi_{}\phi \left(z\right)\left(1-\phi \left(z\right)\right)+\phi \left(z\right)\left(1-\chi_{}\right)$$

which (in contrast with the virial expansion used in Reference [23]) is applicable at arbitrarily large polymer volume fractions up to ϕ(z)≤1.

Combining Equations (2) and (4), we obtain an implicit dependence of the polymer volume fraction ϕ(z) in the brush on *z* as

(5) $$-\ln\left(1-\phi \left(z\right)\right)-2\chi_{}\phi \left(z\right)=\frac{3}{2a^{2}}\kappa^{2}\left(\Lambda^{2}-z^{2}\right)$$

The osmotic pressure inside the brush is given by the following equation:

(6) $$\begin{array}{c} \Pi \left(z\right)=\phi \left(z\right)\frac{\partial f\{\phi (z)\}}{\partial \phi (z)}-f\left\{\phi \left(z\right)\right\}=\\ k_{B}T[-\ln\left(1-\phi \left(z\right)\right)-\phi \left(z\right)-\chi \phi^{2}\left(z\right)] \end{array}$$

The normalisation condition

(7) $${\left(\sigma R\right)}^{-1}\int_{0}^{\min\{R,H\}}\left(R-z\right)\phi \left(z\right)dz=N$$

allows us to find Λ=Λ(R) in the case of “closed” pores or the brush thickness *H* if there is a hollow channel in the pore center, H≤R.

In the case of an “open pore”, H≤R, the polymer volume fraction at the edge of the brush, $\phi \left(H\right)\equiv \phi_{H}$, can be found from the condition of vanishing osmotic pressure, Π(z=H)=0, which leads to an equation for $\phi_{H}$ as a function of $\chi_{}$

(8) $$-\ln\left(1-\phi_{H}\right)-\phi_{H}-\chi_{}\phi_{H}^{2}=0$$

By substituting z=H into Equation (5), we find parameter Λ in an open pore as

(9) $$\Lambda^{2}=H^{2}-\frac{2a^{2}}{3\kappa^{2}}\left[\ln\left(1-\phi_{H}\right)+2\chi_{}\phi_{H}\right]$$

As follows from Equation (8), $\phi_{H}=0$ and, consequently, Λ=H in an open pore at $\chi_{}\leq 1/2$ (i.e., under good or theta-solvent conditions). Under poor solvent conditions, $\chi_{}>1/2$, in the open pore $\phi_{H}>0$ and Λ<H. Under poor solvent conditions, $\phi_{H}$ defined by Equation (8) coincides with the polymer volume fraction in a polymer globule. Remarkably, under poor solvent conditions, $H^{2}-\Lambda^{2}$ is independent of the pore radius *R*.

A hollow channel in the center of the pore appears upon a decrease in the solvent strength or upon an increase in *R* at H(R)=R, where H(R) is calculated for an open pore, H≥R. This is equivalent to the condition Π(z=R)=0, i.e., ${\left[\ln\left(1-\phi \left(z\right)\right)-\chi \phi^{2}\left(z\right)-\phi \left(z\right)\right]}_{z=R}=0$, where the polymer concentration ϕ(z) depends on *R* through Λ(R) calculated from Equation (7).

Under good or theta-solvent conditions, χ≤1/2, the condition of the channel opening can be found directly from the condition ϕ(z=R)=0, that is R=Λ(R)

An analytical solution can be found under good solvent conditions, when $f\left(\phi \left(z\right)\right)/k_{B}T\approx v\phi^{2}\left(z\right)$ with v=1/2−χ. In this case,

(10) $$\phi \left(z\right)=\frac{3}{4v}\kappa^{2}\left(\Lambda^{2}-z^{2}\right)$$

and from Equation (7), we find

(11) $$\Lambda^{2}=\frac{R^{2}}{6}+\frac{4vN}{3\pi \kappa^{2}hR^{2}}$$

so that

(12) $$\phi \left(z\right)=\frac{3}{4v}\kappa^{2}\left(\frac{R^{2}}{6}+\frac{4vN}{3\pi \kappa^{2}hR^{2}}-z^{2}\right)=\frac{3\kappa^{2}}{4v}\left(\frac{R^{2}}{6}-z^{2}\right)+\frac{N}{\pi hR^{2}}$$

where $\frac{N}{\pi hR^{2}}$ is average polymer concentration in the pore. As follows from Equation (12), upon a decrease in the solvent strength (decrease in *v*, brush contraction), the local polymer concentration decreases at $z\geq R/\sqrt{6}$ but increases at $z\leq R/\sqrt{6}$. This applies as long as binary repulsive interactions remain dominant (good solvent conditions).

The condition of the channel opening, Λ=R or ϕ(z=R)=0, leads to

(13) $$R_{opening}={\left(\frac{8vN}{5\pi \kappa^{2}h}\right)}^{1/3}=2{\left(\frac{8v\sigma }{5\pi^{2}\eta^{2}}\right)}^{1/3}N$$

At $R\geq R_{opening}$, there is a hollow channel in the pore center. As follows from Equation (13), $R_{opening}$ is proportional to *N* and increases upon an increase in σ and *v* and decreases upon replacement of linear brush forming chains by branched ones (increase in η).

At $R\geq R_{opening}$, the brush thickness (calculated from Equations (7) and (12)) can be found from the equation

(14) $$H^{4}\left(\frac{8}{3}\frac{R}{H}-1\right)=\frac{16vN\sigma R}{3k^{2}}$$

which in the limit R≫H reduces to

(15) $$H=H_{plan}=2N{\left(\frac{v\sigma }{\pi^{2}\eta^{2}}\right)}^{1/3}$$

which coincides with the result obtained in [23].

Remarkably, as follows from Equations (13) and (15), the ratio

(16) $$\frac{R_{opening}}{H_{plan}}={\left(\frac{8}{5}\right)}^{1/3}>1$$

is independent of topological ratio η and grafting density σ.

Under poor solvent conditions, χ>1, the unconfined brush (H≤R) is collapsed and $\phi \left(z\right)\approx \phi_{H}$, which can be found from Equation (8). The critical pore radius $R_{opening}$ can be estimated from simple packing conditions, $R_{opening}\approx 2N\sigma /\phi_{H}$, which increases as a function of σ and decreases with decreasing solvent strength (an increasing $\phi_{H}$) but, remarkably, is independent of the topology of the bruh forming chains. At $R\leq R_{opening}$, polymer distribution in the closed pore is fairly uniform with the polymer volume fraction ϕ≈2Nσ/R.

### 2.2. Brush Thickness and Pore Opening/Closing Threshold

**Figure 2:**
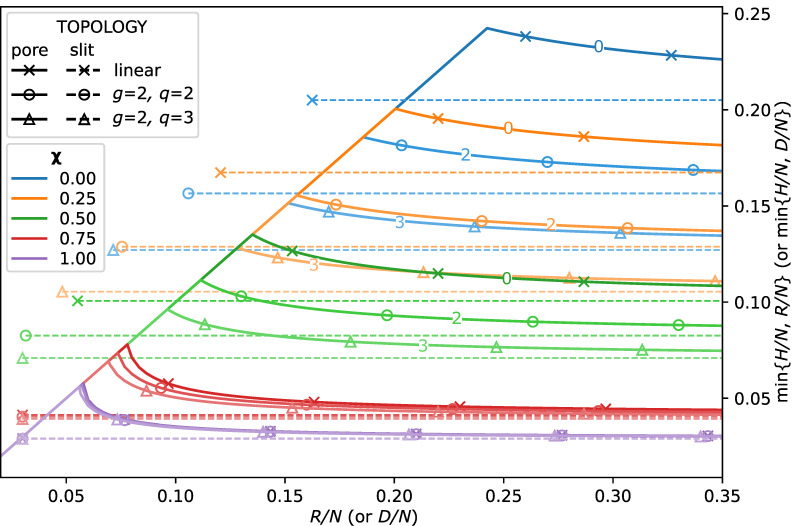
Normalised brush thickness, min{H/N;R/N}, as a function of the normalised pore radius, R/N, for selected values of the χ-parameter (0;0.25;0.5;0.75;1.0) and different topologies (linear and dendritic) of the brush forming macromolecules, as indicated with (g,q) numbers. Horizontal dashed lines correspond to the thickness *H* of a brush grafted onto a planar surface. The corresponding values of χ are color-coded, and topologies are indicated with markers, where cross, circle, and square markers are linear chains and the second generation dendroids g=2 of different branching functionalities q=2 and q=3, respectively. Numbers on the solid lines denote the dendron functionality *q*, where 0 corresponds to linear chains.

In Figure 2, we present the reduced thickness of the brush, min{H,R}/N, as a function of normalised *N* pore radius, R/N, for selected values of the χ-parameter and for three architectures of the brush forming chains: linear and dendrons of the second generation (g=2) with branching functionality q=2 and q=3. The unconfined and confined brush regimes correspond, obviously, to min{H,R}=H and min{H,R}=R, respectively. Horizontal dashed lines correspond to the thickness of the brush (with the same N,σ and χ) grafted onto a planar surface (the limit of R→∞). As one can see from the figure, the thickness H(χ,R) of a unconfined brush in the pore is systematically larger that of the brush grafted to a planar surface and monotonously increases upon a decrease in the pore radius *R*. At H(χ,R)→R, the hollow channel in the pore center vanishes (the closing/opening point), and at smaller *R*, the brush fills the pore. For a given pore radius *R* and solvent strength χ, the thickness H(χ,R) of an unconfined brush of linear chains is larger than that of a dendron brush, whereas for a given *R* and selected topology of the chains, the brush thickness H(χ,R) monotonously decreases as a function of χ (decreasing solvent strength).

**Figure 3:**
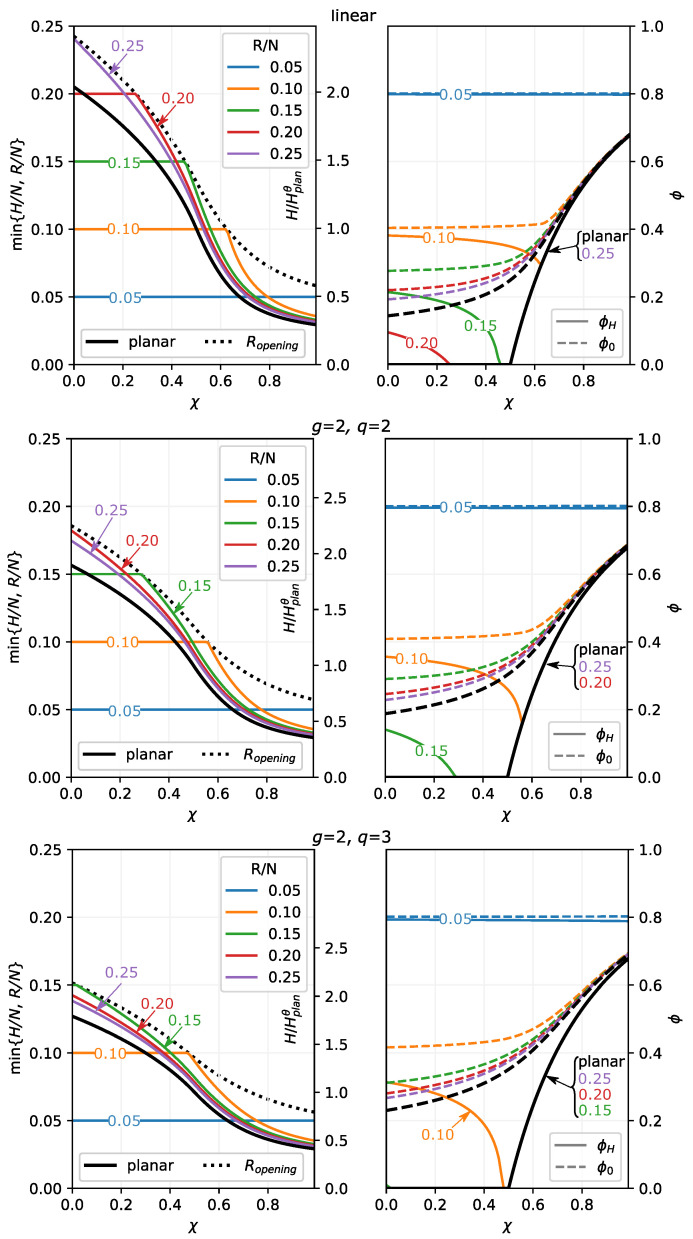
Normalised brush thickness, min{H/N;R/N}, (**left**) and polymer volume fractions at the edge of the brush, ϕ(χ,z=H) (solid lines) and at the grafting surface ϕ(χ,z=0) (dashed lines) as a function of χ-parameter for different pore radius *R* (R=250 (unrestricted); 150; 100; 75; 50) compared with the polymer volume fractions in planar brush with the same N=1000,σ=0.02. The upper, middle, and lower rows correspond to brushes of linear chains, and dendrons of the second generations with functionality q=2 (**middle** row) and q=3 (**lower** row), respectively. The pore radii are universally color-coded through all of the frames. Solid black lines correspond to brushes grafted onto the planar surface. The dotted lines in the left frames trace the dependence $R_{opening}\left(\chi ,g,q\right)$ on χ. Note the secondary axis with brush thickness normalised as $\min\{H/H_{\theta }^{plan};R/H_{\theta }^{plan}\}$.

These trends are also illustrated in Figure 3 where we present the dependence of min{H,R} (left) and polymer volume fractions at the grafting surface, ϕ(χ,z=0), and at the edge of the brush, ϕ(χ,z=min{H,R}), (right) on χ parameter for a set of selected values of the reduced pore radius, R/N, and for three polymer topologies: linear chains (upper row), and dendrons of the second generation with branching functionality q=2 (middle row) and q=3 (lower row). Black solid lines in the left column correspond to the reduced thickness, H(χ,R→∞)/N of the unconfined brush grafted to a planar surface, whereas dotted black line corresponds to the pore opening threshold, H(χ,R)/N=R/N. The smaller the pore radius, R/N, the larger the brush thickness at any given χ. Therefore, the dotted line (R/N at pore opening) lies systematically above the solid line (the planar brush thickness).

As one can see from Figure 3, irrespective of the confinement, the polymer volume fraction close to the grafting surface, ϕ(χ,z=0), is an increasing function, whereas the polymer volume fraction at the edge of the brush, ϕ(χ,z=min{H,R}), is a decreasing function of χ. These trends and the difference between both values become less pronounced (density distribution thoughout the brush becomes more uniform) as confinement becomes stronger or/and degree of branching of the brush forming chains increases (dendron compared to the linear chain brush). For dendron brushes (middle and lower rows in Figure 3), the brush thickness is systematically smaller and the χ-range corresponding to the open pore, H≤R, is more extended than for the brushes of linear chains.

**Figure 4:**
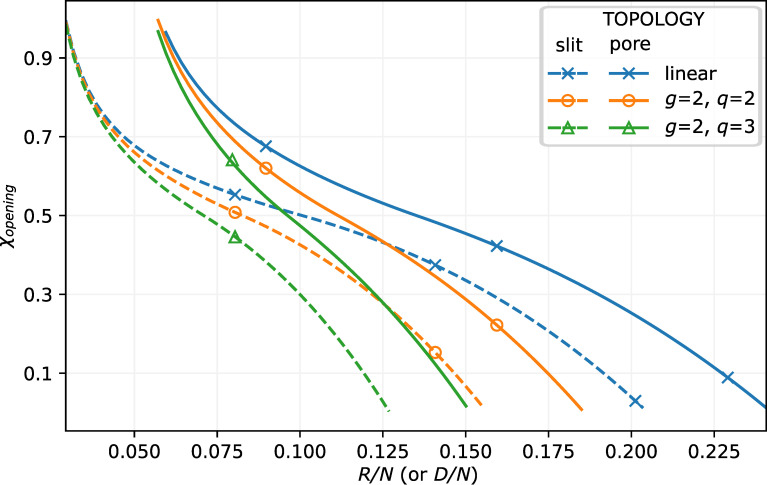
$\chi_{opening}$ for the pore (solid lines) or a slit (dashed lines) with grafted linear, or dendron brush g=2,q=2, or q=3 with N=1000,σ=0.02 as a function of the pore radius *R* or the slit thickness *D* (see Figure 1) normalised by *N*. Topologies are indicated with markers, where cross, circle, and square markers are linear chains and dendroids of the second generation, g=2 of different functionalities q=2 and q=3, respectively.

The dependences of $\chi_{opening}$ corresponding to the pore opening/closing threshold on the pore radius, *R*, (or the slit half-width, *D*) for linear chain and dendron brushes (with the same *N* and σ) are presented in Figure 4. As follows from the figure, at given pore radius *R*, the pore opening occurs at better solvent strength conditions (i.e., at smaller χ) for dendron brushes than for linear chain brushes. For example, the pore opening may occur only under poor solvent conditions for the linear chains brush whereas in the pore decorated by dendrons with the same *N* and σ the central channel emerges already under good solvent conditions. This trend is clearly explained by the weaker swelling of dendron brushes under good or theta-solvent conditions. As expected, the difference between brushes of linear chain and dendron brushes vanishes under poor solvent conditions or confinement (pore filling).

### 2.3. Polymer Density Distribution in the Pore

**Figure 5:**
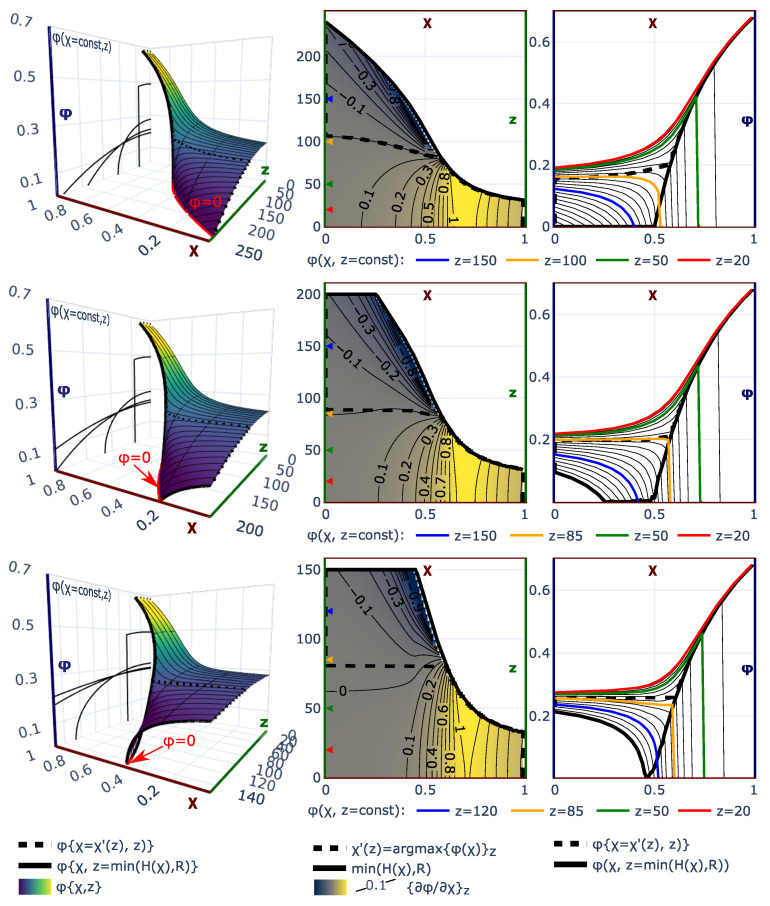
Polymer volume fraction ϕ(χ,z) (left column), contour plot of the derivative ${\left(\partial \phi (\chi ,z)/\partial \chi \right)}_{z}$ in the (χ,z) plain (middle column) and dependence of the local polymer volume fraction ϕ on χ at selected distances *z* from the pore wall (right column) for the brush of linear chains, N=1000,σ=0.02 grafted in cylindrical pore. The rows of the grid correspond to different pore radii: R=250 (unconfined at any χ brush, H≤R, upper row), R=200,150 (confined (pore-filling) under good solvent condition brushes, middle and lower rows). Solid black lines in the left and right columns represent the local polymer volume fraction at the edge of the brush ϕ(χ,z=min{H(χ),R)}); in the middle column, ϕ(χ,z=min{H(χ),R)}) is projected on χ−z plane, thus tracing the dependence of min{H(χ),R)} on χ. Dashed black lines in the middle column correspond to the values of χ for every given *z* that maximises the local polymer volume fraction $\chi^{{}^{\prime }}=\arg\max{\left\{\phi \left(\chi \right)\right\}}_{z}$; corresponding maxima in the polymer volume fraction $\phi (\chi^{{}^{\prime }}\left(z\right),z)$ are plotted with dashed black lines in the left and right columns.

The evolution of the polymer volume fraction distribution across the cylindrical pore upon variations in χ-parameter (solvent strength) is illustrated by Figure 5. The upper row corresponds to the case when the brush is unconfined, H≤R, at any solvent strength. In the middle row, closing/opening of the hollow channel in the center of the pore, H(χ,R)=R, occurs upon a decrease in the solvent strength at χ≤1/2; the lower row depicts the situation when the closing/opening occurs at $\chi_{opening}\approx 1/2$ (close to theta-solvent conditions).

In the left column in Figure 5. we present 3D profiles of the polymer volume fraction ϕ(z,χ) as a function of solvent quality, χ, and distance *z* from the pore wall. At any value of χ, the polymer density profile is a decreasing function of *z* in the range of z∈[0,min{H,R}). In accordance with Equation (8), under good and theta-solvent conditions, χ≤1/2, the polymer volume fraction in a unconfined brush, H≤R, vanishes continuously at the brush edge, z=H, whereas under poor solvent conditions, χ≥1/2, the polymer volume fraction exhibits a finite jump from $\phi_{H}>0$ to zero at the brush edge. The polymer volume fraction at z=R in a confined brush is nonzero at any solvent strength and decreases upon an increase in χ. If the channel opening occurs under good or theta-solvent conditions, $\chi_{opening}\leq 1/2$, then the polymer volume fraction at the edge of the brush, z=H≤R, vanishes continuously in the range of $\chi_{opening}\leq \chi \leq 1/2$. Otherwise, if $\chi_{opening}\geq 1/2$, the nonzero polymer volume fraction is found at the edge of the brush, ϕ(z=min{H,R}), in both confined and non-confined regimes.

The dependence of the polymer volume fraction ϕ(χ,z) on χ exhibits qualitatively different patterns depending on the selected distance *z* from the pore wall. In the right column of Figure 5, the volume fraction ϕ(χ,z) is plotted as a function of χ for a few selected values of *z*; the black solid lines correspond to the polymer volume fraction at the edge of the brush, z=min{H,R}. In the middle column of Figure 5, the contour plots of the derivative ${\left(\partial \phi (\chi ,z)/\partial \chi \right)}_{z}$ are presented. As one can see from these plots, the polymer volume fraction ϕ(χ,z) monotonously increases as a function of χ and asymptotically approches unity at χ→∞ for $z\leq H_{min}$, where $H_{min}=H\left(\chi \to \infty \right)=R\left(1-\sqrt{1-R_{min}/R}\right)$ is the thickness of fully collapsed, ϕ=1 brush and $R_{min}=2N\sigma$ is the minimal radius of the pore accomodating the brush with given *N* and σ. At larger distances from the surface, $H_{min}\leq z\leq H^{\theta }$, where $H^{\theta }=H\left(\chi =1/2\right)$ is the brush thickness in the theta-point, the polymer volume fraction increases as a function of χ as long as $\chi \leq \chi^{*}\left(z\right)$, where the value $\chi^{*}\left(z\right)$ is found from the condition $H(\chi^{*}\left(z\right))=z$, and then drops down to zero. At even larger distance from the grafting surface, $z\geq H^{\theta }$, the polymer volume fraction ϕ(χ,z) exhibits more complex behaviours as a function of χ, i.e., it either monotonously decreases upon an increase in χ if *z* is close to the periphery of the brush or passes though a maximum and then continously vanishes.

### 2.4. Numerical Self-Consistent Field Theory: Beyond Analytical Strong-Stretching Approximation

We note that the analytical SS-SCF approach employed above for calculating the polymer volume fraction ϕ(z) and the brush thickness in the open pore, H≤R, does not account for Gaussian fluctuations of the non-stretched terminal segments of the brush forming chains. These fluctuations give rise to the decaying “tail” in polymer density distribution protruding beyond the brush edge, i.e., to non-vanishing polymer concentration at z≥H. Due to these density fluctuations, the effective radius r=R−H of the hollow chanel is slightly smaller than predicted by SS-SCF theory.

The width of the tail is expected to vary non-monotonously as a function of solvent strength (χ): in the regimes of good and theta solvent, χ≤1/2, it increases upon a decrease in the brush thickness *H* caused by decreasing solvent strength as [31]

(17) $$\xi ≅\frac{N^{2/3}}{H^{1/3}\left(\chi \right)}$$

because of the decreasing overall stretching of the chains in the brush. Below the theta point, i.e., at χ≥1/2, polymer density fluctuations and extension of the tail in the polymer density distribution are controlled by the thermal correlation length [32], which scales as

(18) $$\xi_{t}∼{\left(\chi -1/2\right)}^{-1}$$

close to the theta-point and further decreases upon an increase in χ≥1. Therefore, the relative width of the fluctuating tail scales as $\xi /H∼N^{-1/3}$ under good and theta-solvent conditions and as $\xi_{t}/H∼N^{-1}$ under poor solvent conditions.

**Figure 6:**
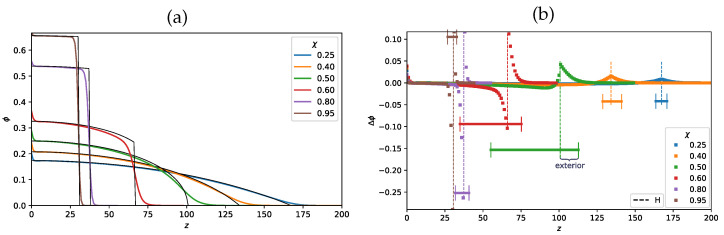
Polymer volume fraction profile calculated by the numerical SF-SCF method $\left(\phi_{SF}\right)$ and within analytical SS-SCF approximation $\left(\phi_{SS}\right)$ for selected values of χ parameter, (0.25;0.40;0.50;0.60;0.80;0.95) for N=1000,σ=0.02, planar surface, R=∞ (**a**) The difference, $\Delta \phi \left(z\right)=\phi_{SF}\left(z\right)-\phi_{SS}\left(z\right)$, between numerically and analytically calculated polymer density profiles for the same selected values of the χ-parameter (**b**). Here, *z* is a distance from the grafting surface or the number of the layer for lattice SF-SCF model. In panel (**b**) the brush thickness calculated by analytical method is indicated by dashed vertical lines, and horizontal bars shows the total range ϵ of Gaussian fluctuations, separated by the dashed vertical line into interior and exterior regions of the brush.

**Figure 7:**
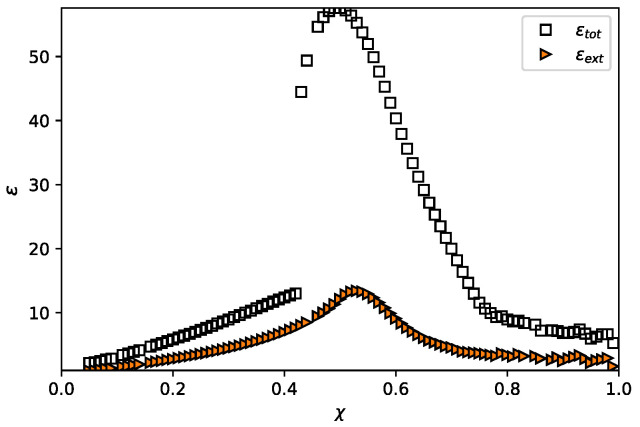
Width of the range of Gaussian fluctuations of the non-stretched terminal segments of the chains (square marker). and the size of the tail in the polymer density profile protruding above z=H (triangle marker) as a function of χ-parameter for the brush of linear chains, grafted to a planar surface, R=∞ for N=1000,σ=0.02.

In order to obtain a more detailed description of the polymer density distribution near the edge of the brush, we used a numerical modelling approach based on the self-consistent field Scheutjens–Fleer method [33,34]. The latter method does not involve any approximations concerning the degree of stretching of the brush forming chains and accounts for the Gaussian fluctuations of the non-stretched terminal segments of the chains. The polymer volume fraction profiles calculated for a planar (R→∞) brush under varied solvent strength conditions analytically (SS-SCF approximation) and numerically using the SF-SCF scheme and their difference, $\Delta \phi \left(z\right)=\phi_{SF}\left(z\right)-\phi_{SS}\left(z\right)$ are presented in Figure 6. As one can see from Figure 6, |Δϕ(z)| vanishes far from the edge of the brush but is maximal at z=H. The shape of the Δϕ(z) curves changes with the variation in the solvent strength: they are fairly symmetric under good or poor solvent conditions and asymmetric close to the theta point. In agreement with presented above analytical estimations, the magnitude and the width of Δϕ(z) increase when the solvent strength decrease from good to theta-solvent conditions, reach maxima in the theta-point, and decrease upon further increase in χ in the range of poor solvent. This is illustrated in Figure 7, where the width of the Δϕ(z) curves is plotted as a function of χ (with the same values of N=1000,σ=0.02). As seen from Figure 7, The width of Δϕ(z) curve for a dendron brush is expected [13] to be smaller than that for the brush of linear chains at any χ. This observation brings us to the conclusion that the “tail” in the polymer volume fraction profile is most extended around the theta-point and is more pronounced for the brushes of linear chains rather than for dendron brushes.

Ending this section, we have to point out a certain peculiarity in the pore closing/opening transition when it occurs under poor solvent conditions. According to the SS-SCF approximation, under poor solvent conditions, χ≥1/2, the polymer density in the open pore, H≤R, exhibits a discontinuity at the edge of the brush, at z=H. The polymer volume fraction at the edge of the brush, ϕ(z=H), obeys Equation (8) and coincides with the equilibrium volume fraction in a polymer globule [32]. The unfavourable contacts between monomer units and poor solvent at the interface between the collapsed brush and the surrounding solution give rise to excess free energy $\gamma k_{B}T$ per unit area of the interface. In the vicinity of the theta point, (χ−1/2)≪1, the surface tension γ scales as [32] $\gamma ∼{\left(\chi -1/2\right)}^{2}$, whereas far from the theta-point, at χ≥1, the linear grouth, γ∼χ, is predicted with a smooth crossover between these two asymptotics in the χ∼1 range [35]. In the case of the open cylindrical pore, this excess interfacial free energy produces a negative Laplace pressure −2γ/(R−H). In order to eliminate this unfavourable interface by closing the pore and thus to vanish the excess interfacial free energy, the brush may undergo additional swelling with respect to its equilibrium thickness calculated according to the SS-SCF scheme. Consequently, the closing of the pore is expected to occur, when the channel radius is r=R−H≪R, as a jump-like (first order) transition. This transition occurs when the free energy corresponding to the equilibrium thickness of the brush calculated above within SS-SCF scheme and complemented by the excess free energy of the brush-solvent interface (per chain), $F_{surf}/k_{B}T≅\gamma h\left(R-H\right)≅\gamma s\left(R-H\right)/R$, becomes equal to the free energy, corresponding to the filling the pore overstretched brush. Mathematically, in the “pore closing” transition range, the free energy exhibits a local minimum corresponding to the open pore and an edge minimum corresponding to the close pore, and the minima are separated by the free energy barrier.

Assuming that r=R−H≪R and $H≅R≅N/s\phi_{H}$, where the collapsed polymer volume fraction $\phi_{H}=\phi_{H}\left(\chi \right)$ is given by Equation (8), we find that the minimal equilibrium radius of the open channel scales as

(19) $$r_{min}=R-H≅{\left(\frac{\gamma N^{2}\sigma^{2}}{\phi_{H}^{3}u\left(\chi \right)}\right)}^{1/3}∼N^{2/3}$$

The derivation of Equation (19) and expression for u(χ) are presented in the Appendix A. Following from Equation (19),

(20) $$\frac{r_{min}}{R}∼N^{-1/3}$$

Hence, for sufficiently long chains, N≫1, and wide pores, the relative minimal width of the hollow channel at which jump-wise closing of the pore occurs is negligible compared with the pore radius.

Obviously, since the contribution of conformational entropy to the overall free energy of the collapsed brush under poor solvent conditions is negligible, the above estimate of the jump-wise opening/closing transition point is fairly insensitive to the topology (linear or dendritic) of the brush forming chains.

**Figure 8:**
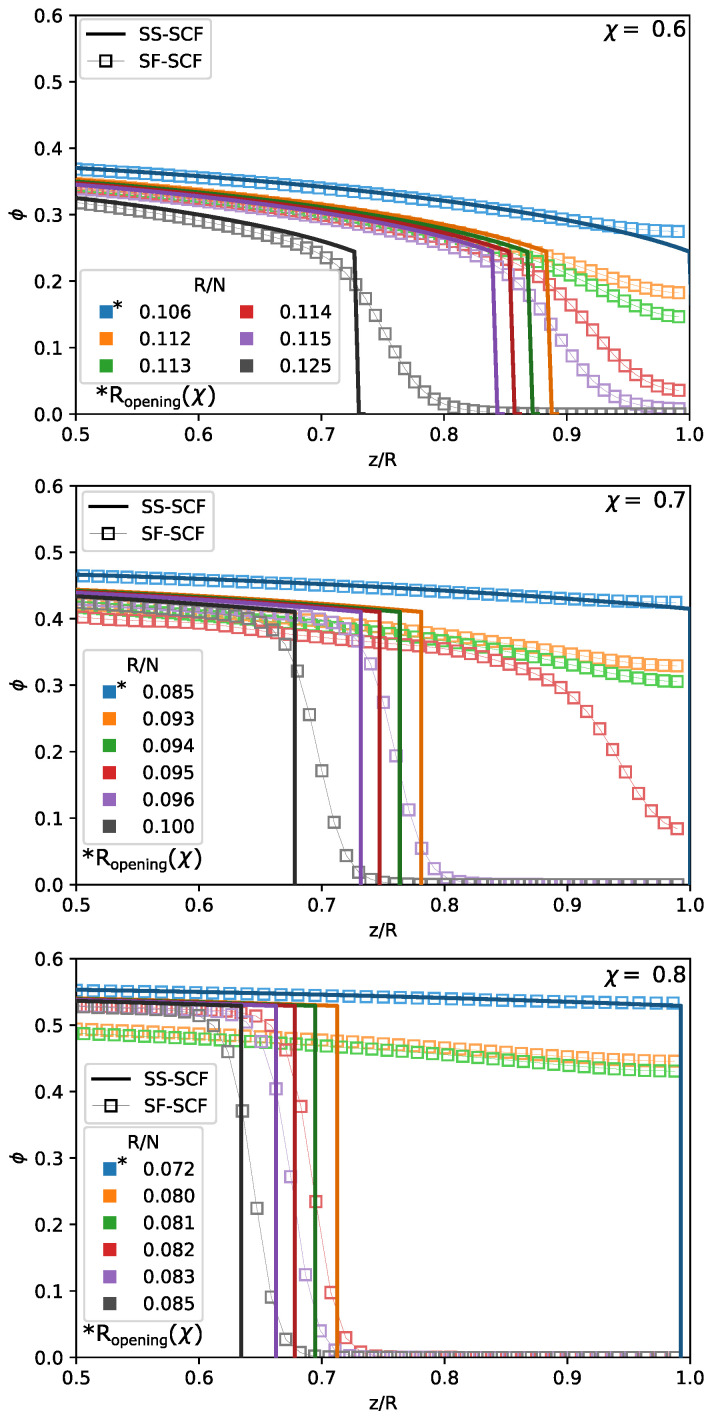
Polymer density profiles ϕ(χ,z/R) as a function of normalised distance from the pore wall z/R for selected values of χ parameter (0.6,0.7,0.8) corresponding to three frames from the top to bottom for selected values of pore radii, including $R_{opening}\left(\chi \right)$ calculated within SS-SCF approximation and marked by an asterix. Pore radii are color-coded, and the density profiles calculated by numerical SF-SCF method $\left(\phi_{SF}\right)$ are presented as squared markers while solid lines trace the polymer density profiles $\left(\phi_{SS}\right)$ calculated analytically. N=1000,σ=0.02.

The evidence for the jupm-wise pore opening/closing transition is provided by mumerical SF-SCF calculations. In Figure 8, the polymer volume fraction profiles ϕ(z/R) calculated analytically and numerically under poor solvent conditions (χ=0.6;0.7 and 0.8) are plotted for variable pore radius R/N close to the predicted by analytical theory pore opening/closing threshold, $R\approx R_{opening}\left(\chi \right)$. As follows from the Figure 8, at $R\leq R_{opening}$, the pore is filled with polymer (no hollow channel) and the numerically calculated profile perfectly matches the analytical one. If the pore radius $R\gg R_{opening}$ and there is a hollow channel in the pore center with ϕ(z)=0, the numerically caclulated profile is slightly more extended than the analytical one due to Gaussian fluctuations of the terminal chain segments, as discussed above. However, close to the analytically caclulated transition point, $R\geq R_{opening}$, numerical calculations show that the pore is still closed with relatively high polymer concentration in the pore center, at z=R, whereas according to analytical theory they should be a hollow channel in the pore center. Moreover, according to numerical calculations, the shape of the polymer density profiles abruptly changes (the pore opsning/closing transition) within a very narrow range of variation of *R* around $R_{opening}^{*}$, where $R_{opening}^{*}$ evaluated from numerical calculations numerically is larger than $R_{opening}$ calculated analytically.

**Figure 9:**
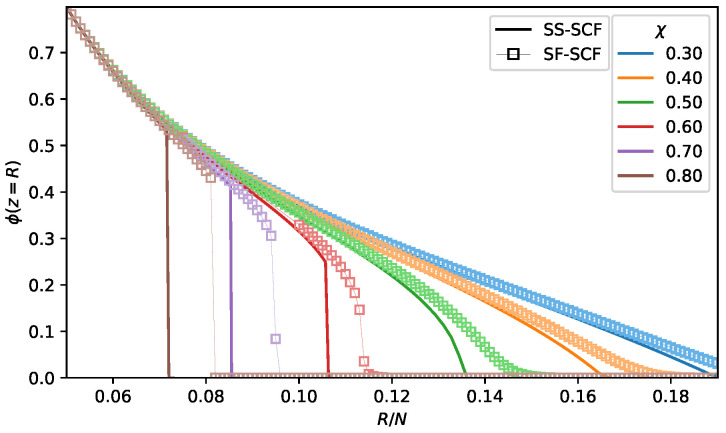
Local polymer density in the center of a pore ϕ(z=R) as a function of normalised pore radius R/N for selected values of χ-parameter (0.3,0.4,0.5,0.6,0.7,0.8). The value of χ are color coded, and the results calculated by numerical SF-SCF method $\left(\phi_{SF}\left(z=R\right)\right)$ are presented as squared markers while solid lines trace the values of $\left(\phi_{SS}\left(z=R\right)\right)$ calculated analytically. N=1000, σ=0.02.

The same trend is illustrated by Figure 9, where the polymer volume fraction in the pore center, ϕ(z=R), is plotted as a function of the pore radius *R* for a few selected values of the χ-parameter. As expected, the polymer volume fraction in the pore center monotonously decreases upon an increase in the pore radius *R*. Under good and theta-solvent conditions, χ≥0.5, the polymer volume fraction in the pore center smoothly vanishes above the pore openning threshold, at $R\geq R_{opening}$. The numerical calculations predict slightly larger pore radius corrersponding to the opening transition than the analytical theory due to Gaussian fluctuations of the terminal chain segments. Under poor solvent conditions, analytical theory predicts a drop in the polymer density at the edge of the brush upon the pore opening, which is in agreement with numerical results. However, numerical results indicate that this drop in the polymer density (the pore opening) emergies at significantly larger pore radius $R=R_{opening}^{*}>R_{opening}$, than predicted by analytical theory.

**Figure 10:**
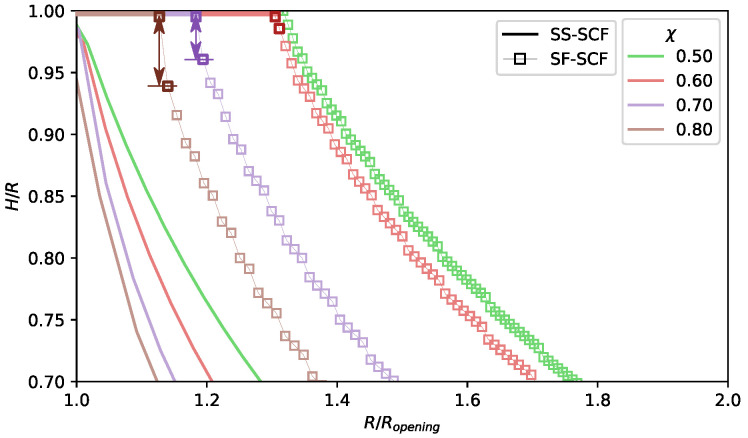
Polymer brush thickness min{H,R} in a poor solvent as a function of the normalised pore radius $R/R_{opening}$ calculated using the numerical SF-SCF method (squares) and within analytical SS-SCF approximation (solid lines) for selected values of χ parameter, (0.50;0.60;0.70;0.80) for N=1000,σ=0.02. Here, $R_{opening}$ is the minimal open pore radius calculated within analytical SS-SCF approximation. Note the jumps (darkened color arrows) for χ=0.8 and χ=0.7 between close sequential calculations with varied *R*; the upper darkened marker on a scatter correspond to the closed pore H/R=1, while the lower darkened marker correspond to open pores with brush height H<0.95R for χ=0.8.

In Figure 10, the ratio between the brush thickness *H* (calculated analytically or numerically) and the pore radius *R* normalised by $R_{opening}$ is plotted for a set of the χ-values corresponding to theta and poor solvent conditions. The brush thickness is a decreasing function of *R* (at given χ) or a decreasing function of χ at given $R/R_{opening}\left(\chi \right)$. The analytical curves cross at $H\left(\chi ,R\right)/R=R/R_{opening}=1$ since, by definition, $H\left(\chi ,R_{opening}\right)=R_{opening}$. The numerically calculated dependences behave differently: the arrows in Figure 10 indicate jump-wise closing-opening of the pore under poor solvent conditions upon continuous variation in the pore radius *R*, the magnitude of the jump increases upon an increase in χ.

## 3. Cluster Formation and Longitudinal Versus Lateral Instability of the Collapsed Brush in the Pore

### 3.1. Clusters in a Brush Confined in a Slit

It is known that a planar polymer brush collapsed in poor solvent retains a laterally uniform structure as long as the brush thickness is larger than the Gaussian size of an individual polymer coil, $H\geq N^{1/2}$. The SS-SCF formalism is applicable for description of the brush structural properties under poor solvent conditions pre-assuming lateral brush uniformity only if the condition $H\geq N^{1/2}$ is fulfilled. A further decrease in the solvent strength provokes splitting of laterally uniform brush into clusters (“pinned micelles”) [36].

Each cluster consists of a collapsed globular core with radius $R_{c}$ connected to the grafting points of the cluster-forming chains by extended “legs” of length *L*, which serves as a characteristic size (“footprint”) of the cluster in the lateral direction. The cluster size is related to the number *p* of chains per cluster (aggregation number) as $L≅{\left(ps\right)}^{1/2}$.

Below, we consider brushes formed by linear polymer chains in the regime of moderately poor solvent strength conditions, τ≡|χ−1/2|≤1, when the volume fraction of monomer units in the collapsed core ≃τ, while surface tension γ at the polymer-solvent interface is related to τ as $\gamma ≃k_{B}T\tau^{2}$. We consider the case when the surface tension γ at the solvent–polymer interface is equal to the difference Δγ between surface tensions at the polymer–surface and solvent–surface interfaces. In this case, the globular core of pinned micelle acquires a perfectly spherical shape. The structural properties of clusters were derived in References [36,37] in the form of scaling dependences as

(21) $$p≅N^{4/5}\tau^{2/5}s^{-3/5}$$

(22) $$L≅{\left(\tau N^{2}\right)}^{1/5}s^{1/5}$$

(23) $$R_{c}≅N^{3/5}{\left(\tau \right)}^{-1/5}s^{-1/5}$$

and all of the numerical pre-factors of the order of unity are omitted here and below.

Decomposition of a laterally uniform collapsed brush with the polymer volume fraction ϕ≅τ and thickness H≅N/sτ into clusters occurs at $\tau ≅N^{1/2}/s$ when $L≅R_{c}≅N^{1/2}$. (Obviously, at sufficiently high grafting density, $s\leq N^{1/2}$, the brush retains a laterally uniform structure up to fully collapsed, ϕ≅1, state.) An increase in τ (decreas in the solvent strength) in the range $\tau \geq N^{1/2}/s$ leads to an increase in the number of chains per cluster and lateral cluster size (Equations (21) and (22)) with concomitant decrease in the size of the globular core $R_{c}$ (Equation (23)). The latter trend is explained by increasing core density.

We now consider a slit-like (planar) pore with polymer brushes grafted onto both upper and lower surfaces and decomposed into clusters (which is the case at $\tau \geq N^{1/2}/s$). As long as the half-distance between the grafting surfaces *D* exceeds the cluster size *L*, each cluster comprises the chains originating from the same surface. However, when the slit width becomes smaller that the span of an individual cluster, that is at D≤L, clusters belonging to the upper and the lower surfaces merge. As a result, at $R_{c}\leq D\leq L$, each sluster comprises approximately equal number of chains originating from upper and lower surfaces, whereas the aggregation number *p*, lateral cluster size *L*, and core size $R_{c}$ still follow the sacling laws given by Equations (21)–(23).

Upon a further decrease in the distance 2D between the pore walls, the cores of the clusters become compressed at $D\leq R_{c}$, where $R_{c}$ is given by Equation (23). The cores of compressed clusters acquire the shape of the “pancakes” with the radius $\rho_{c}$, thickness *D*, and total surface area $A=\pi \rho_{c}^{2}+2\pi \rho_{c}D$. The volume *V* of the pancake core with $\rho_{c}\gg D$ yields

(24) $$V\approx \pi \rho_{c}^{2}D≃\frac{pN}{\tau }$$

to give

(25) $$\rho_{c}≃{\left(\frac{pN}{\tau D}\right)}^{1/2}$$

and surface area per chain

(26) $$\frac{A}{p}≃\frac{N}{\tau D}+{\left(\frac{a^{3}ND}{\tau p}\right)}^{1/2}$$

Although the first term in Equation (26) is dominant, it does not depend on aggregation number *p* and can be omitted from further consideration. Moreover, a possible difference between γ and Δγ does not affect the second, *p*-dependent term in Equation (26).

The aggregation number in such clusters can be found from minimisation of the free energy (per chain)

(27) $$F_{cluster}/p=\tau^{3/2}{\left(ND/p\right)}^{1/2}+p^{1/2}\tau s^{1/2}$$

where the first term describes the *p*-dependent interfacial free energy $∼\tau^{2}A/p$ and the second term accounts for the free energy of stretched legs. As a result of the minimisation with respect to *p*, we obtain

(28) $$p≅{\left(\tau ND/s\right)}^{1/2}$$

(29) $$L≅{\left(\tau NDs\right)}^{1/4}$$

(30) $$\rho_{c}≅N^{3/4}{\left(\tau Ds\right)}^{-1/4}$$

**Figure 11:**
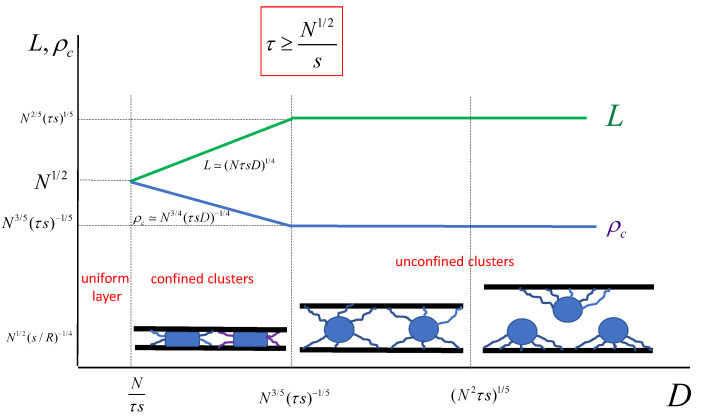
Dependence of the cluster lateral dimension, *L*, and cluster core radius in the lateral direction, $\rho_{c}$ on the thickness of the slit-like pore *D*. The regimes of unconfined (free) and confined clusters are indicated.

**Figure 12:**
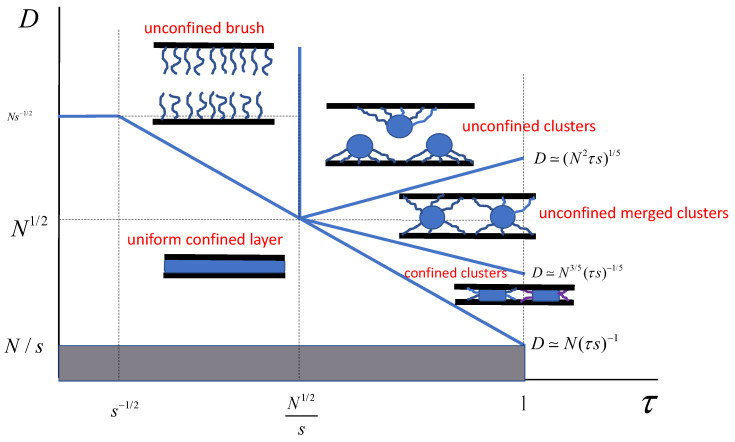
Diagram of states of the brush compressed in a planar slit-like pore under poor solvent conditions in the pore width *D*, - solvent strength, and τ coordinates.

Hence, a decrease in *D* leads to a decrease in *p* and *L* with concomitant increase in the pancake radius $\rho_{c}$. When, upon a decrease in *D*, the span of the cluster *L* and the core radius $R_{c}$ become equal, $L≅\rho_{c}$, the brush acquires a laterally uniform structure. In Figure 11, we present the evolution of *L* and $\rho_{c}$ as a function of *D*. Remarkably, in the regime of clusters made from polymers grafted to both upper and lower surfaces with unconfined cores, $R_{c}\leq D\leq L$, there is no dependence of the cluster properties *L* and $R_{c}$ on the slit width *D*. In Figure 12, the diagram of states of the compressed brush under poor solvent conditions is presented.

### 3.2. Clusters in a Brush Confined in the Cylindrical Pore

Similar to the planar brush case (corresponding to the R→∞ limit), the brush grafted onto the inner walls of a cylindrical pore with radius $R\geq N^{1/2}$ splits at $\tau \geq N^{1/2}/s$ into clusters attached to the pore walls. Structural properties of the clusters evolve upon an increase in τ according to Equations (21)–(23) as long as the cluster size remains smaller than the pore radius, L≤R (quasi-planar regime). At $\tau ≅R^{5}/N^{2}s$, or equivalently, at pore radius,

(31) $$R=R_{*}≃{\left(s\tau N^{2}\right)}^{1/5},$$

the cluster size *L* becomes comparable with the pore radius *R*, indicating the lower limit of quasi-planar regime.

**Figure 13:**
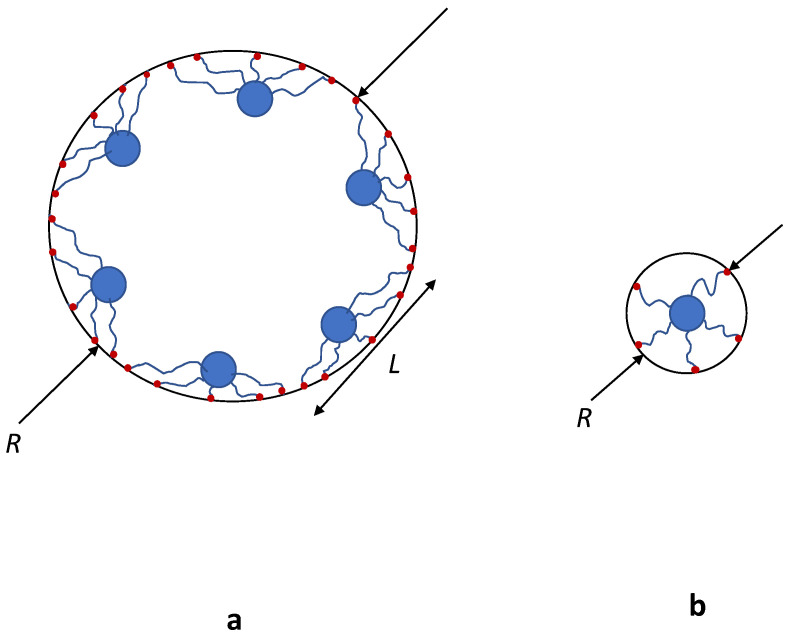
Schematics of clusters pinned in quasi-two dimensional (**a**) and quasi-one-dimensional (**b**) regime in a cylindrical pore.

A decrease in the core radius $R≲R_{*}$ leads to rearrangement of clusters along the pore in a quasi-one-dimensional array (see Figure 13). Let h≅s/R be the axial distance per grafted chain in the pore and L≅ph be the longitudinal dimension of a cluster. The aggregation number in the one-dimentional array of clusters is then found from minimisation of the free energy (per chain)

(32) $$F_{cluster}/p=\tau^{4/3}N^{2/3}p^{-1/3}+\tau p\cdot \left(s/R\right)$$

which leads to

(33) $$p≅N^{1/2}\tau^{1/4}\cdot {\left(R/s\right)}^{3/4}$$

(34) $$L≅N^{1/2}\tau^{1/4}\cdot {\left(s/R\right)}^{1/4}$$

(35) $$R_{c}≅N^{1/2}\tau^{-1/4}\cdot {\left(s/R\right)}^{-1/4}$$

In scaling terms, the structural properties of clusters in the one-dimensional regime follow the same dependences as derived for clusters attached to a thin cylinder in Reference [38].

**Figure 14:**
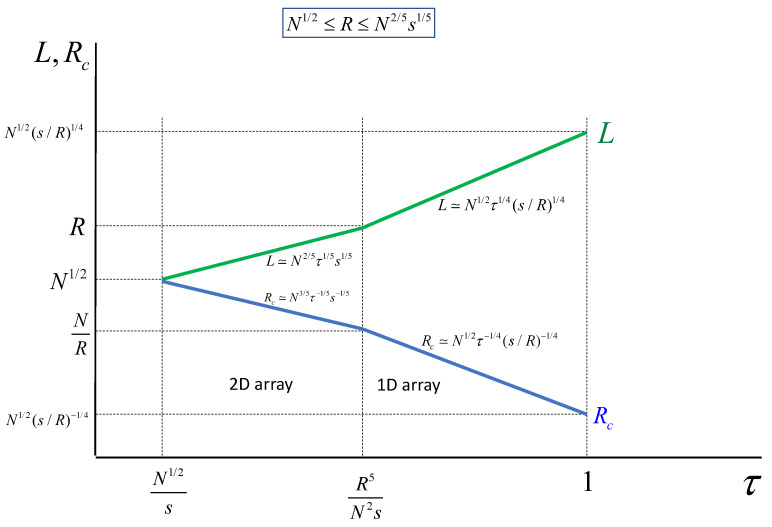
Dependence of the cluster size, *L*, and cluster core radius, $R_{c}$ on solvent strength τ≅(χ−1/2) at pore radius $N^{1/2}\leq R\leq N^{2/5}s^{1/2}$, and grafting density is $\sigma =1/s\leq N^{-1/2}$.

The evolution of the cluster size *L* and cluster core dimensions $R_{c}$ as a function of τ are presented in Figure 14. Obviously, the transition from quasi-planar to quasi-one-dimensional regimes occurs at $\tau ≅R^{5}/N^{2}s$ only if the pore radius $N^{1/2}\leq R\leq N^{2/5}s^{1/5}$. In the case $R\geq N^{2/5}s^{1/5}$, the cluster remain quasi-planar up to τ≅1.

## 4. Discussion and Conclusions

In the present paper, the theory of conformational transitions in polymer brushes grafted to the inner surface of a cylindrical or slit-like pore was developed using analytical methods based on the strong-stretching self-consistent field (SS-SCF) approximation. The theory applies to brushes made from linear chains as well as dendrons and cycled [39] chains. The topology of the brush forming chains is taken into account within a universal formalism through the so-called topological ratio, which quantifies the degree of branching and affects the magnitude of the self-consistent field potential in the brush. The Flory approximation was used to describe the solubility of monomer units, which made it possible to describe both strongly swollen and completely collapsed brushes inside a pore within the framework of a unified theory. The density distribution of the polymer across the pore was obtained as a function of the thermodynamic quality of the solvent, the degree of chain polymerisation, and the grafting density. It was shown that the dependence of the local concentration of the polymer in the pore on the solvent strength (quantified by the Flory–Huggins χ parameter) has a significantly different character at different distances from the pore wall, namely, monotonically increases (near the pore wall), and it monotonically decreases (at the edge of the brush) or passes through a maximum in the intermediate region. This result is important for predicting the distribution of the flux of diffusing particles across the pore upon a change in the solvent strength.

The effect of surface curvature on the threshold solvent quality at which a hollow channel opens in the center of the pore is analysed, and it is shown that, at the same values of the grafting density and degree of polymerisation, in the case of a cylindrical pore, this occurs under conditions of poorer solvent than in the case of a slit-like pore. It was also shown that replacing linear chains by dendrons decorating the pore wall leads to a more uniform distribution of monomer units across the pore, but the pore opening threshold is shifted towards better solvent conditions.

According to the SS-SCF approach, the pore closing occurs progressively when the equilibrium thickness, *H*, of the brush reaches the pore radius, *R*. However, under poor solvent conditions, a jump-wise (first order) closing-to-opening transition may occur in a cylindrical pore at R−H≪R. The metastability of the narrow open channel of radius r=R−H≪R arises due to excess free energy (surface tension) of the curved interface between the edge of the collapsed concave cylindrical brush and filling the pore poor solvent. As demonstrated by scaling arguments, the minimal open channel radius scales as $r_{min}∼N^{2/3}$. Hence, this transition might be observable for relatively narrow pores (short chains), whereas for wide pores, $r_{min}/R∼N^{-1/3}$ and the relative width of the minimal open channel is negligibly small. We remark that an alternative mechanism of the pore closing at H≤R may involve long-wave instability of longitudinally uniform polymer density distribution, which is beyond the scope of our current analysis.

The analytical theory was complemented by calculations based on the numerical Scheutjens–Fleer self-consistent field method. This method does not involve any pre-assumption of strong stretching of the brush forming chains and allowed us to analyse deviations in the polymer density profiles near the edge of the brush from those predicted by SS-SCF analytical theory. It was demonstrated that the width of the fluctuating “tail” in the polymer density profiles protruding beyond the edge of the brush (as defined analytically) varies non-monotonically as a function of the solvent strength, i.e., it passes through a maximum near the theta-point and decreases towards both good and poor solvent conditions. Under good and theta-solvent conditions, the “tail” is formed by non-stretched terminal segments of the chains, whereas under poor solvent conditions, the “tail” length is proportional to the thermal blob size.

Microphase segregation, which occurs under poor solvent conditions and leads to the disintegration of a longitudinally (or laterally) homogeneous brush into finite-size clusters under spatial constraints imposed by the walls of the pore to which the polymer brush is grafted, was studied using the scaling approach for the particular case of linear brush forming chains. The formation of clusters (“pinned micelles”) consisting of a spherical globular core connected by stretched segments (legs) to grafting points of brush forming chains has been previously described for free (non-constrained) polymer brushes attached to planar [36] or convex [40,41] surfaces. In the case of a wide pore, the clusters have the same (quasi-planar) structure regardless of the pore geometry. However, we predicted qualitatively different scenarios of cluster transformation in a narrow slit-like or cylindrical pore.

When the width of the slit-like pore decreases to a size smaller than the lateral size (footprint) of a cluster, the clusters may include chains grafted to the opposing walls of the slit. A further decrease in the slit width results in the confinement of the globular cores of the clusters, and the core acquires the shape of an oblate cylinder (pankace) with a base radius exceeding the slit width. A further decrease in the slit width is accompanied by an increase in the lateral size of the cluster core and a decrease in the number of chains included in the cluster and, therefore, a decrease in the lateral size of the cluster as a whole, up to the formation of a laterally homogeneous collapsed layer. In the case of a cylindrical pore, a decrease in the pore radius down to the size of the cluster leads to a change in symmetry and the transformation of a quasi-two-dimensional array of clusters into a quasi-one-dimensional one. In this case, the cores of the clusters are arranged along the axis of the pore with a periodicity equal to the longitudinal size of the cluster. At a fixed grafting density (pore surface area per chain), the aggregation number and the size of the globular core of the cluster decrease with decreasing pore radius, while the longitudinal size of the cluster increases. It can be hypothesised that, under the conditions of the formation of a quasi-one-dimensional system of clusters, the diffusive transport of sufficiently small solutes through a cylindrical pore occurs predominantly in the near-wall region where the polymer density is reduced.

The results obtained in the present paper concerning the evolution of the radial distribution of the polymer density in the pore upon variation of the solvent strength or architecture (degree of branching) of the decorating pore macromolecules can be applied for the formulation of principles of control of selective diffusive transport through polymer-modified pores, which is the scope of a forthcoming publication. 

## Data Availability

Not applicable.

## Appendix A. Pore Opening/Closing Threshold: Calculation of *r_min_*

In this appendix, we present the calculation of the minimal radius $r_{min}$ of the hollow channel in the pore center under poor solvent conditions, χ≤1/2, below which a jump-wise closing of the pore, r→0, takes place.

As under poor solvent conditions the brush is collapsed and the polymer volume fraction in the unconfined brush is approximately constant, $\phi \left(\chi ,z\right)\approx \phi_{H}$, where $\phi_{H}=\phi_{H}\left(\chi \right)$ is defined by Equation (8), we can introduce the minimal pore radius accomodating unconfined brush as

(A1) $$R_{0}=\frac{2N\sigma }{\phi_{H}}$$

which approximately coincides with $R_{opening}\left(\chi \right)$ calculated within SS-SCF scheme.

Assume that a jump-wise closing of the pore caused by a negative Laplace pressure occurs when the pore radius is slightly larger than $R_{0}$, i.e., at $R=R_{0}\left(1+\delta \right)$, where δ≪1, which corresponds to the radius of the hollow channel $r=R_{0}{\left[{\left(1+\delta \right)}^{2}-1\right]}^{1/2}≅\sqrt{2\delta }R_{0}$ and brush thickness $H=R-r\approx R_{0}\left(1-\sqrt{2\delta }\right)$.

In order to close the pore and thus to eliminate the brush–solvent interface, the brush has to stretch beyond its equilibrium thickness, H(χ,R)<R up to H=R which leads to a decrease in the average polymer volume fraction in the brush from $\phi \approx \phi_{H}$ to $\phi \approx \phi_{H}\left(1-2\delta \right)$. The corresponding increase in the voume contribution to the free energy of the brush (due to a decreased number of favourable under poor solvent conditions monomer–monomer contacts) can be estimated as

(A2) $$\Delta F_{vol}/k_{B}T=N\left[\left(\frac{f(\phi )}{\phi }\right)-{\left(\frac{f(\phi )}{\phi }\right)}_{\phi =\phi_{H}}\right]$$

where f(ϕ)/ϕ is the free energy per monomer unit. By expanding Equation (A2) in powers of $\phi -\phi_{H}$ (that is, in powers of δ≪1) and keeping in mind that ${\left[\partial \left(f\left(\phi \right)/\phi \right)/\partial \phi \right]}_{\phi =\phi_{H}}=0$, we arrive at

(A3) $$\Delta F_{vol}/k_{B}T≅Nu\left(\chi \right)\delta^{2}$$

where

(A4) $$u\left(\chi \right)=2\frac{\partial^{2}}{\partial \phi^{2}}{\left(\frac{f(\phi )}{\phi }\right)}_{\phi_{H}}\phi_{H}^{2},$$

we note that u(χ)≥0 since the free energy of the brush exhibits a minimum at $\phi =\phi_{H}$.

The excess volume free energy of the overstretched brush has to be balanced by the gain in the interfacial free energy due to the vanishing brush–solvent interface,

(A5) $$\Delta F_{surf}/k_{B}T=2\pi rh\gamma \approx 2^{1/2}\gamma \sigma^{-1}\delta^{1/2}$$

where we used for the channel radius $r≅\sqrt{2\delta }R_{0}$. We disregard the conformational entropy penalty for additional stretching of the chains upon channel closing because the entropic contribution to the free energy is negligible in the collapsed under poor solvent conditions brush.

By equating $\Delta F_{vol}=\Delta F_{surf}$ given by Equations (A3) and (A5), respectively, we arrive at

(A6) $$\delta ={\left(\frac{\sqrt{2}\gamma }{\sigma Nu(\chi )}\right)}^{2/3}$$

which allows us to calculate the minimal radius of the hollow channel at which jump-wise closing of the pore occurs as

(A7) $$r_{min}=R_{0}\delta^{1/2}=\frac{2^{7/6}\gamma^{1/3}N^{2/3}\sigma^{2/3}}{\phi_{H}u^{1/3}\left(\chi \right)}$$

in accordance with Equation (19).
